# The Bergen Facebook addiction scale: a reliability generalization meta-analysis

**DOI:** 10.3389/fpsyg.2024.1444039

**Published:** 2025-01-07

**Authors:** Jian-Ling Ma, ZhengCheng Jin, Chang Liu

**Affiliations:** ^1^Chongqing University of Posts and Telecommunications, Chongqing, China; ^2^Yangtze Normal University, Fuling District, China

**Keywords:** Facebook addiction, Facebook addiction scale, reliability, reliability generalization, meta-analysis

## Abstract

The Bergen Facebook addiction scale (BFAS) is a screening instrument frequently used to evaluate Facebook addiction. However, its reliability varies considerably across studies. This study aimed to evaluate the reliability of the BFAS and its adaptation, the Bergen Social Media Addiction Scale (BSMAS), and to identify which study characteristics are associated with this reliability. We performed a reliability generalization meta-analysis involving 173,641 participants across 127 articles, which reported 147 Cronbach’s alpha values for internal consistency. The random-effects model revealed that the pooled Cronbach’s alpha values were 0.8535 (95% CI [0.8409, 0.8660]) for the BFAS and 0.8248 (95% CI [0.8116, 0.8380]) for the BSMAS. Moderator analyses indicated that the mean and standard deviation of the total scores accounted for 10.06 and 36.7% of the total variability in the BFAS alpha values, respectively. For the BSMAS, the standard deviation of the total scores and sample size accounted for 13.54 and 10.22% of the total variability alpha values, respectively. Meta-ANOVA analyses revealed that none of the categorical variables significantly affected the estimated alpha values for either the BFAS or BSMAS. Our findings endorse the BFAS and BSMAS as reliable instruments for measuring social media addiction.

## 1 Introduction

Social media addiction is a psychological condition characterized by an excessive focus on social media platforms. Individuals with this addiction feel a strong compulsion to use social media and invest substantial time and energy into it, often at the expense of their social activities, learning, interpersonal relationships, mental health, and overall well-being (Andreassen and Pallesen, 2014). Research has consistently highlighted the detrimental health effects of social media addiction, including sleep disturbances (Ho, 2021; Marino et al., 2018), impaired decision-making (Delaney et al., 2018), and increased risk of depression (Ho, 2021; Mamun and Griffiths, 2019; Seabrook et al., 2016). Therefore, accurately assessing social media addiction is crucial for understanding its underlying mechanisms and potential harmful effects. The Bergen Facebook addiction scale (BFAS; Andreassen et al., 2012) is a widely utilized tool for assessing Facebook addiction. It is a self-report scale designed primarily for college students and is based on six criteria: salience, tolerance, mood modification, relapse, withdrawal, and conflict, as defined by Brown (1993) and Griffiths (1996). The BFAS includes a 6-item short version and an 18-item standard version. Each item is rated on a 5-point Likert scale (1 = very rarely, 5 = very often). The total score is calculated by summing individual item scores, with higher scores indicating greater levels of Facebook addiction. The higher the total score, the more severe the addiction to the Facebook platform. Preliminary findings indicate that the BFAS demonstrates good validity. Total BFAS scores correlate well with other measures of Facebook activity, neuroticism, and extraversion, and show a negative relationship with conscientiousness. Additionally, higher BFAS scores are associated with delayed sleep onset and wake times (Andreassen et al., 2012). Given the proliferation of social media platforms beyond Facebook, researchers have adapted the BFAS to assess addiction across various platforms through the Bergen Social Media Addiction Scale (BSMAS; Schou Andreassen et al., 2016). Both the BFAS and BSMAS have been translated into several languages, including German (Brailovskaia et al., 2018), Spanish (Elphinston et al., 2022), Portuguese (da Veiga et al., 2019), and Chinese (Yam et al., 2019), due to their demonstrated validity.

In classical test theory, reliability refers to how consistently a measurement tool produces results. It is typically defined as the ratio of true score variance to the total variance, reflecting the proportion of variance in scores due to the true score rather than measurement error (Higgins et al., 2003; Rodriguez and Maeda, 2006). Cronbach’s alpha is commonly used to assess reliability because it provides a measure of internal consistency, indicating how well the items in a scale measure the same underlying construct. Researchers frequently use it as the reliability indicator for the BFAS. The BFAS itself has a good Cronbach’s alpha (0.83). Studies using the BFAS have also found high internal consistency reliability in specific contexts. However, there are several issues with reporting this reliability indicator in studies. For the BFAS, Cronbach’s alpha ranges from 0.66 (Błachnio et al., 2017; Brailovskaia et al., 2023) to 0.94 (Satici, 2019; Soraci et al., 2023). Similarly, for BSMAS, it varies from 0.66 (Chung et al., 2019) to 0.92 (Brailovskaia et al., 2019; Hoşgör et al., 2021). These variations highlight significant discrepancies in reported reliability. Another major issue is that, when some studies have used the BFAS or BSMAS, they report reliability values from previous research rather than calculating them from their own data, which can lead to inaccurate or misleading conclusions. This phenomenon of omitting or improperly reporting reliability values is an issue of reliability induction (Henson and Thompson, 2002). It is clear that reliability is context-dependent and can vary based on sample and testing conditions. Discrepancies in reliability estimates as well as reliability induction threaten the reliability of statistical analyses and research conclusions based on such indicators. Therefore, although the BFAS and BSMAS are widely used, no study has systematically explored the variability in the reliability of the two tools in different test scenarios and estimated their overall reliability.

Reliability generalizability analysis is a method that evaluates the average reliability of a measurement tool, explores variability in reliability across studies, and identifies factors that affect reliability (Henson and Thompson, 2002; Vacha-Haase, 1998). This study uses this approach to address gaps in the current research on the BFAS and BSMAS. This study aims to (1) estimate the average internal consistency reliability of the BFAS and BSMAS, (2) assess the variability in reliability across different studies, (3) identify research characteristics that might influence reliability, and (4) address issues related to reliability induction.

## 2 Materials and methods

The review methods and reporting followed the Reliability Generalization Meta-analysis (REGEMA) guidelines (Sánchez-Meca et al., 2021), which outline best practices for conducting reliability generalization studies. The research protocol was registered with the International Prospective Register of Systematic Reviews (PROSPERO ID: CRD42021295390) to ensure transparency and adherence to systematic review standards.

### 2.1 Study search strategy

Systematic searches were conducted in the EBSCO, Elsevier, Springer, ProQuest, Wiley Online Library, and CNKI databases using keywords such as ‘Facebook addiction,’ ‘social media addiction,’ and related terms. No search limits were applied. In addition, backward searches were performed from recent qualitative reviews and key studies to identify additional relevant articles. The final search was completed on December 30, 2021.

### 2.2 Study selection criteria

**Figure 1:**
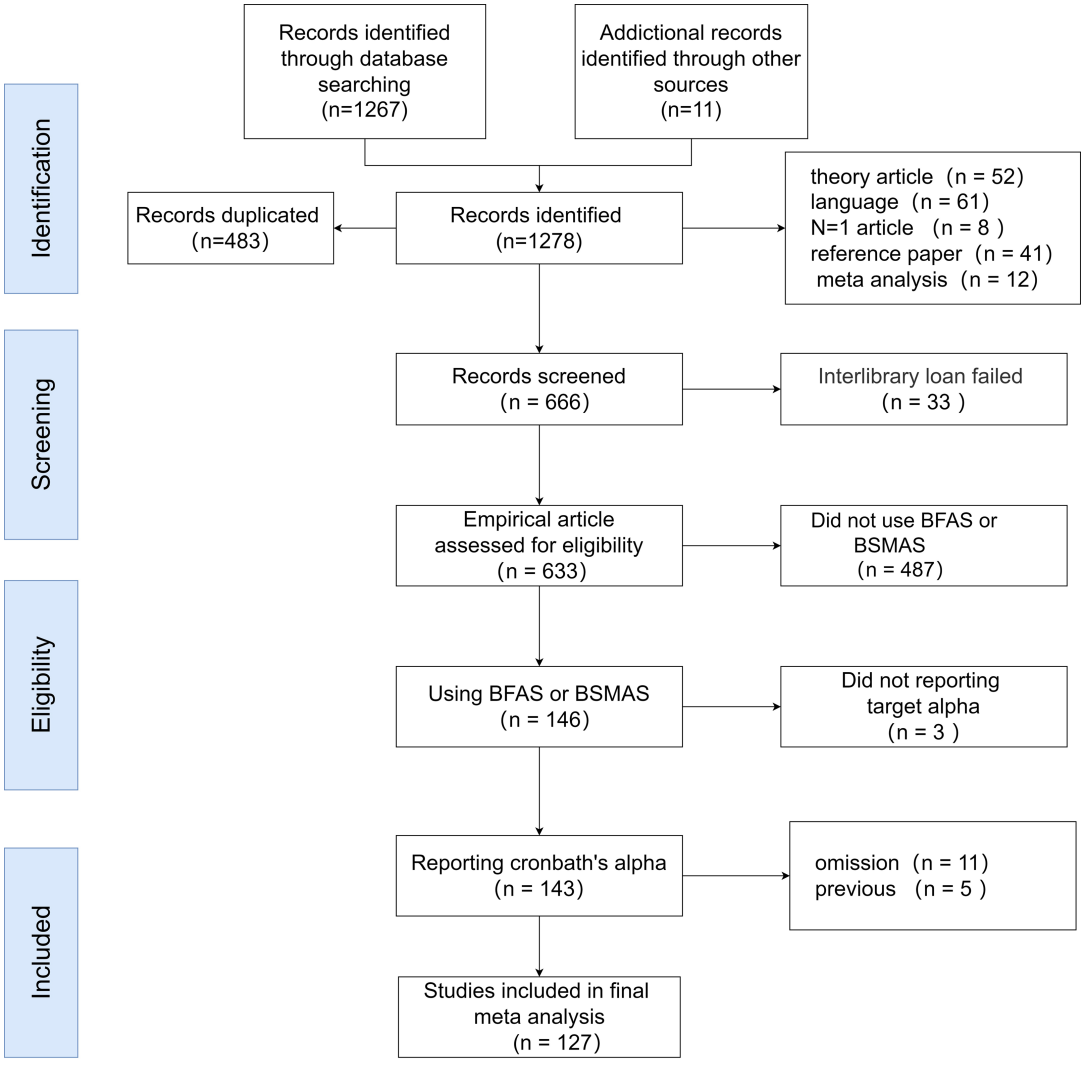
Flowchart of selection and inclusion of articles.

To be included in this reliability generalization meta-analysis, studies had to meet the following criteria: (1) Published in English or Chinese; (2) Empirically reported Cronbach’s alpha values for the scales used; (3) Published in a peer-reviewed academic journal or as a dissertation to ensure quality. Figure 1 illustrates the study selection process.

### 2.3 Data extraction and coding

Characteristics were only extracted from studies that reported the target Cronbach’s alpha values. To examine how study characteristics influenced alpha values, the following category moderators were coded: COVID-19, administration, country, test language, study aim, study nature, test length, participant group, sampling method, and social media platform. Continuous variables included publication year, sample size, mean and standard deviation of sample age, female proportion, and mean and standard deviation of the total score. Missing data for studies was recorded as such, and no imputation was performed. The coding manual, developed by the first and second authors, included detailed guidelines for extracting and categorizing study characteristics. The coding process involved dual coding of a random sample of 40 studies to ensure accuracy. Disagreements between coders were assessed using the intraclass correlation coefficient (ranging from 0.99 to 1) for continuous variables and kappa coefficients (ranging from 0.87 to 1.00) for categorical variables.

### 2.4 Data analysis

The current study used Cronbach’s alpha values for the reliability generalization meta-analysis. The transformation method was not employed based on recommendations by Thompson and Vacha-Haase (2000), who suggested that it may not be necessary for the analyses conducted. Random-effect models using a frequentist framework were chosen for their ability to account for variability between studies, in line with standard practice in meta-analysis. Inverse variance was used as the weighting method. The between-study variance estimator is a restricted maximum likelihood method. The confidence limits of the overall reliability estimates were computed using the method proposed by researchers (Knapp and Hartung, 2003). Heterogeneity was assessed using the Q test and the I^2^ index, which indicates the percentage of total variation across studies due to heterogeneity rather than chance (Thompson and Vacha-Haase, 2000). I^2^ values of 25, 50, and 75% correspond to low, moderate, and high heterogeneity, respectively (Higgins et al., 2003). To explain the variance of alpha values, moderator analysis was applied. Specifically, meta-analyses of variances (meta-ANOVA) and meta-regression analyses were applied for categorical and continuous variables, respectively. Moreover, the adjustments method proposed by Knapp and Hartung were used (Knapp and Hartung, 2003) to examine the statistical significance of the moderator variable and explain the residual heterogeneity. The *Q*_W_ and *Q*_E_ indices were used to examine model misspecification of meta-ANOVA and meta regression, respectively. Furthermore, the present study also employed *R*^2^ as an index to quantify the degree of variance explained by the moderator variables. If more than one moderator contributed to the variance of the coefficient alpha, a multiple meta-regression analysis was conducted to identify the unique contributions of the moderators.

Publication bias was assessed using funnel plots for both BFAS and BSMAS, and the trim-and-fill method (Duval and Tweedie, 2000) was used to estimate and adjust for any asymmetry in the funnel plots. The asymmetry of the funnel plots indicates that there are potential coefficient alphas that were not included in the current meta-analysis, and the number of these coefficient alphas can be estimated using the trim-and-fill method. The fail-safe number (Durlak and Lipsey, 1991) was calculated to assess the robustness of the meta-analytic findings against publication bias. Meta-analytic results were considered reliable if the fail-safe number exceeded the critical value of 5 × k + 10, where k represents the number of studies included in the analysis. If the fail-safe number falls below this critical value, publication bias or file drawer problems may exist.

All statistical analyses were performed using the metafor (Viechtbauer, 2010) package (V3.8) in the R program 4.1.2. for Windows.

## 3 Results

### 3.1 Description of sample

In total, 127 articles, 147 reliability values, and 173,641 subjects were included in the formal meta-analysis (reliability-induced articles were excluded). Among the 127 articles that reported the target reliability values, the distribution of the number of reliability values in the top five countries were: China (21), Germany (20), the United States (19), Italy (18) and Turkey (11); Concerning the language of the scale, the top five languages were English (38), Chinese (20), German (20), Italian (18) and Turkish (11), and the distribution of the number of subjects was: Norway (47,283), China (46,712), Italy (11,435), the United States (10,137), and Hungary (4073). From the perspective of the language used in the scale, the top five languages in terms of the number of subjects were English (70221), Chinese (45797), Italian (11435), Hungarian (7043), and German (6840). From the perspective of sample distribution, the mixed sample, undergraduate student sample, unknown sample, and adult sample were 96,756, 35,871, 34,600, 3,499, and 2,915, respectively. The number of subjects using the BFAS was 122,018, and the number using the BSMAS was 51,623.

Sixteen articles had reliability-induction issues. Specifically, 12 studies using BFAS had been induced, eight studies omitted reports, and four studies introduced initial reliability. The BSMAS had four studies that had been induced, three studies omitted reports, and one study introduced initial reliability.

### 3.2 Overall reliability estimates and test of heterogeneity

The averaged estimated Cronbach’s alpha value is 0.8407 (95% CI [0.8313, 0.8500]) without considering the version of BFAS and BSMAS; more specific results are presented in Table 1. To further investigate the sources of heterogeneity in the overall reliability estimates, we first analyzed the moderating effect of the version; the results showed that the moderating effect of the version was significant, *Q*_M_ = 10.03, *p* < 0.0001, *Q*_E_ = 5851.985, *p* < 0.0001, τ^2^ = 0.0027, *R*^2^ = 7.04%. The high heterogeneity observed indicates substantial variability in reliability estimates across different studies, suggesting that factors such as sample characteristics and study conditions significantly influence reliability. The overall heterogeneity of each version was tested to further investigate the heterogeneity of the reliability values of different versions. The estimation results are presented in Table 1.

**Table 1:** Mean alpha reliability of BFAS and BSMAS and Heterogeneity analysis results.

			95%CI				
	*n*	α+	LL	UL	τ^2^	*Q*	*I*^2^ (%)
Total	147	0.8407	0.8313	0.8500	0.003	5852.7054***	98.84
BFAS	82	0.8535	0.8409	0.8660	0.003	2934.9131***	97.95
BSMAS	65	0.8248	0.8116	0.8380	0.002	2917.0719***	99.04

*n* = number of Cronbach’s alpha coefficients; α+ = estimated average alpha value; H^2^ = sampling variability index; Tau^2^ = estimated total heterogeneity; *Q* = heterogeneity statistics in the distribution of the Cronbach’s alpha. *I*^2^: total heterogeneity index. ***p < 0.001.

The meta-analysis revealed that the BFAS and BSMAS had high internal consistency, with Cronbach’s alpha values of 0.85 and 0.82, respectively, indicating strong reliability. The results also indicate heterogeneity in the reliability values of both the BFAS and BSMAS; therefore, further analyses are required.

### 3.3 Moderation analysis

#### 3.3.1 Meta-ANOVA for category variables

The summary results of the meta-ANOVA for the categorical variables are shown in Table 2. For all category variables, the estimated average alpha values of the BFAS and BMAS were not statistically significant. For COVID-19, administration, country, test language, study aim, study nature, test length, participant group, sampling method, and social media platform, none of the variables exerted an effect on the average internal consistency reliability of the BFAS and BSMAS. Tables 3 present the reliability estimates between the different levels of the category variables.

**Table 2:** Meta-ANOVA for category variables.

moderator	BFAS			BSMAS		
	*R* ^2^	*F* values	*p*	*R* ^2^	*F* values	*p*
COVID-19	0.000	0.1275	0.7220	0.000	0.0431	0.8362
Administration	0.0123	0.9998	0.3977	0.000	1.0456	0.3916
Country	0.0626	1.1402	0.3350	0.0982	1.5716	0.1173
Test language	0.0000	0.9785	0.4947	0.0747	1.5000	0.1543
Study aim	0.0000	0.4171	0.5203	0.0000	0.0102	0.9898
Study nature	0.0000	0.0014	0.9701	0.0000	1.0319	0.3136
Test length	0.0000	0.7980	0.3744	-	-	-
Participant group	0.0000	0.5398	0.6565	0.000	0.2115	0.8800
Sampling method	0.0157	1.4653	0.2305	0.000	0.9129	0.4401
Social media platform	0.0000	0.2597	0.8542	0.0551	2.5412	0.0870

*R2 = proportion of variance explained by the moderator variable; F values = test of moderators; p = p value for the statistical tests of F values.*

**Table 3:** Comparison of coefficient alpha estimates of the different levels of category variables.

Moderator	BFAS			BSMAS		
	*n*	α+	95%CI	*n*	α+	95%CI
COVID-19						
No	80	0.8538	[0.8410,0.8666]	56	0.8242	[0.8098,0.8385]
Yes	2	0.8394	[0.7601,0.9187]	9	0.8281	[0.7930,0.8632]
Administration						
Online + Paper-pencile	1	0.8100	[0.6980,0.9220]	1	0.7700	[0.6649,0.8751]
NR	11	0.8401	[0.8052,0.8751]	13	0.8310	[0.8009,0.8610]
Online	45	0.8615	[0.8444,0.8786]	38	0.8309	[0.8137,0.8480]
Paper-pencile	23	0.8417	[0.8183,0.8652]	11	0.8000	[0.7665,0.8334]
Telephone	—	—	—	1	0.8000	[0.6943,0.9057]
Study aim						
Correlation	79	0.8542	[0.8414,0.8670]	60	0.8247	[0.8107,0.8388]
Experiment	3	0.8297	[0.7554,0.9040]	3	0.8271	[0.7588,0.8953]
Psychometric	—	—	—	1	0.8180	[0.7108,0.9252]
Study nature						
Applied	76	0.8534	[0.8402,0.8665]	58	0.8271	[0.8132,0.8410]
Confirm	6	0.8543	[0.8082,0.9004]	7	0.8050	[0.7638,0.8461]
Test length						
Long version	5	0.8752	[0.8252,0.9252]	—	—	—
Short version	77	0.8520	[0.8390,0.8650]	65	0.8248	[0.8116,0.8380]
Participant group						
Adolescents	8	0.8517	[0.8111,0.8922]	8	0.8153	[0.7783,0.8524]
Adults	2	0.8401	[0.7590,0.9211]	2	0.8450	[0.7723,0.9177]
Mixed	38	0.8594	[0.8403,0.8784]	33	0.8275	[0.8093,0.8457]
Undergraduate	30	0.8416	[0.8205,0.8628]	18	0.8264	[0.8013,0.8515]
Sampling method						
Convience	61	0.8527	[0.8383,0.8671]	53	0.8206	[0.8059,0.8353]
NR	7	0.8263	[0.7819,0.8706]	1	0.8300	[0.7234,0.9366]
Random	9	0.8550	[0.8174,0.8926]	7	0.8550	[0.8159,0.8941]
Snow balling	5	0.8965	[0.8460,0.9469]	4	0.8226	[0.7702,0.8750]
Social media platform						
Facebook	55	0.8538	[0.8381,0.8695]	2	0.8210	[0.7497,0.8923]
Instagram	3	0.8440	[0.7776,0.9104]	1	0.7000	[0.5882,0.8118]
Snap chat	1	0.9000	[0.7869,1.0131]	—	—	—
Social media	23	0.8516	[0.8279,0.8753]	62	0.8268	[0.8137,0.8400]

#### 3.3.2 Meta-regression for continuous variables

As shown in Table 4, the mean and standard deviation of the total score for BFAS account for 10.06 and 36.7% of the variance of alpha values, respectively. Together, these two variables explained 66.57% of the variance in alpha values. For the BSMAS, the standard deviation of the total score and sample size accounted for 13.54 and 10.22% of the variance in alpha values, respectively. However, these two variables explain only 8.51% of the variance.

**Table 4:** Results of simple meta-regression analysis by the continuous moderator variables.

Moderator	*n*	*b*	*Q*_M_	*p*	*R* ^2^	*Q*_E_
BFAS						
Publication year	82	−0.0033	0.8094	0.3710	0.0000	2819.2302****
Mean of age	72	0.0008	0.6733	0.4147	0.0017	2613.9072****
SD of age	71	0.0021	1.1027	0.2973	0.0081	2527.3068****
Female proportion	72	−0.0006	0.2098	0.6484	0.0000	2720.5324****
Mean of total score	33	0.0027	4.2112	0.0487	0.1006	1245.9475****
SD of total score	33	0.0142	16.8116	**0.0003**	0.3670	961.4841****
Sample size	82	0.0000	1.0619	0.3059	0.0084	2203.5410****
Mean of total score + SD of total score	33	-	25.8499	**0.0001**	0.6657	590.3401****
BSMAS						
Publication year	65	−0.0064	1.7842	0.1864	0.0217	2785.5586****
Mean of age	60	0.0008	0.7624	0.3862	0.0039	2485.9618****
SD of age	60	0.0030	3.1002	0.0836	0.0360	2584.7461****
Female proportion	59	0.0706	1.8243	0.1821	0.005	2419.3051****
Mean of total score	35	−0.0092	3.5271	0.0692	0.0624	606.2396****
SD of total score	35	0.0446	6.4296	**0.0161**	0.1354	905.6529****
Sample size	65	0.0000	6.7404	**0.0117**	0.1022	1755.9959****
SD of total score + sample size	46	-	2.3875	0.1039	0.0851	618.0904****

*n* = number of the Cronbach’s alpha coefficient for each subgroup of moderator variable; *b* = unstandardized regression coefficient; *Q*_M_ = significance test of moderator regression coefficient; *p* = *p* value of significance test; *R*^2^ = total amount proportion of variance accounted for; *Q*_E_ = statistic for test of residual heterogeneity. ****p < 0.0001. Bold values indicate that the *p* values is significant.

### 3.4 Publication bias

**Figure 2:**
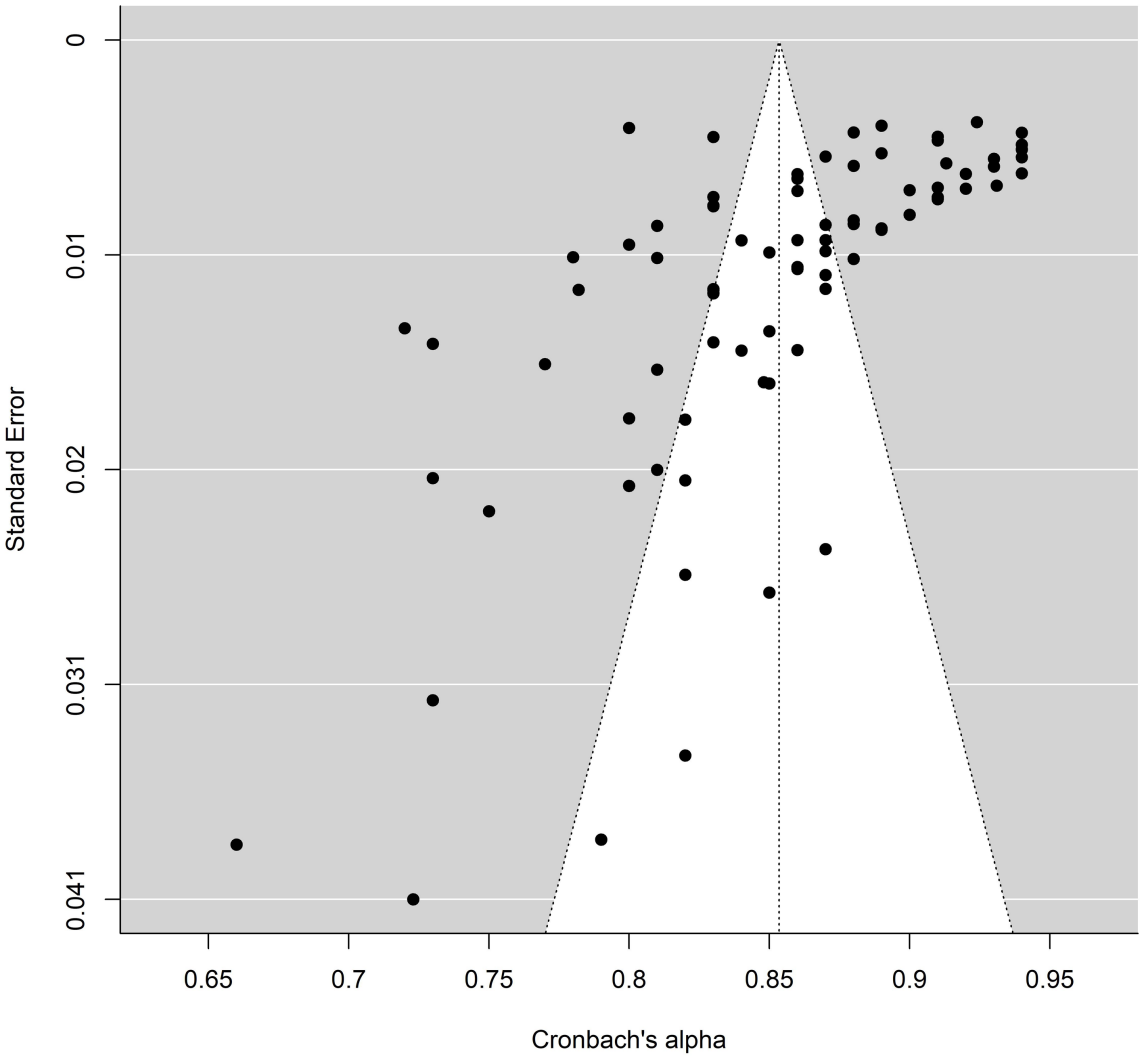
Funnel plot of BFAS.

**Figure 3:**
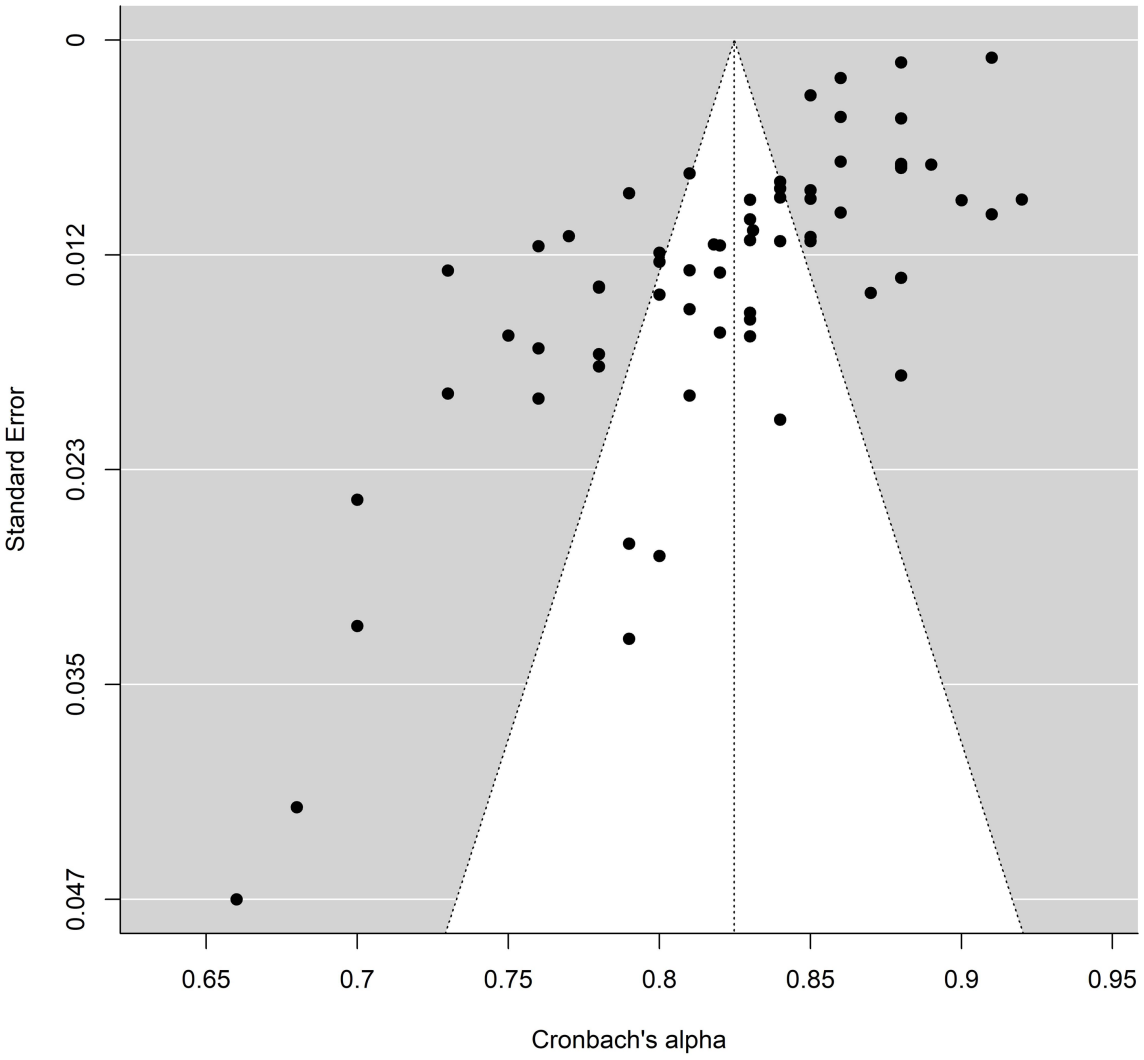
Funnel plot of BSMAS.

To investigate the publication bias of the BFAS and BSMAS, corresponding funnel plots were drawn; the results are shown in Figures 2, 3. The trim-and-fill method results showed that the number of studies on right-side BFAS trimming was zero (SE = 5.1902), indicating no publication bias; for BSMAS, the number of studies on right clipping was zero (SE = 4.6142), indicating no publication bias. The BFAS internal consistency reliability measurements for the Rosenthal (24,493,065), Owen (82), and Rosenberg (22,335,449) methods were calculated for this study; the BFAS values for the methods were 21,193,629 (Rosenthal), 65 (Owen), and 44,612,242 (Rosenberg), indicating that the reliability generalization results are relatively reliable.

## 4 Discussion

The purpose of this study was to conduct a meta-analysis of the BFAS and BSMAS’s internal consistency reliability using a reliability generalization method. The study found that the internal consistency reliabilities of the BFAS and BSMAS were 0.8535 (95% CI [0.8409, 0.8660]) and 0.8248 (95% CI [0.8116, 0.8380]), respectively. Second, there was high heterogeneity between the BFAS and BSMAS studies. Third, Category variables did not significantly moderate the reliability of either scale. However, for the BFAS, the mean and standard deviation of total scores were significant moderators, whereas for the BSMAS, only the standard deviation of total scores and sample size played a role. Fourth, reliability-induction issues were noted in both scales. For the BFAS, eight studies failed to report reliability values, and four studies reported initial reliability values incorrectly. For the BSMAS, three studies omitted reliability reporting, and one incorrectly reported initial values.

According to the evaluation criteria for internal consistency reliability; 0.9 indicates good internal consistency reliability, above 0.8 is ideal, above 0.7 is recommended for modification, and below 0.7 should be reworked (Sijtsma, 2009). This study’s results show that both BFAS and BSMAS have an estimated reliability of more than 0.8, which is ideal. This finding is consistent with the initial reliability values of the two measures, indicating that this tool is reliable for measuring an individual’s social media addiction. Moreover, there was a significantly high heterogeneity between the studies using the two scales. This indicates that there are significant differences in research tools across a wide range of populations, samples, age groups, countries, regions, and publication years. Despite this, the average estimation reliability of the two tools still reached a high level, indicating the stability of the measurement results of the two tools from a side perspective. However, the BSMAS is more heterogeneous than the BFAS, which may be related to the measured social media platforms. The BFAS was specifically designed to investigate Facebook platform addiction, whereas the BSMAS measures a wider range of platforms.

In addition to examining the average estimation reliability and heterogeneity of the BFAS and BSMAS, this study examined the impact of research characteristic variables (continuous and category variables) on their reliability. The results showed that, for the BFAS, the moderating effect of category variables on the average estimate of reliability was not statistically significant, while the mean and standard deviation of the test scores affected its reliability. For the BSMAS, only the standard deviation of the test scores and sample size affected its internal consistency reliability. The year that the study was published, mean age of the subjects, standard deviation of the subjects’ age, proportion of women in the sample, and total test score had no impact on internal consistency. For the BFAS, the mean and standard deviation of the test scores independently explained about 10.06 and 36.7% of the variance, respectively, while the two together explained about 66.57% of the variance. For the BSMAS, the standard deviation of test scores and sample size explained approximately 13.54 and 10.22% of the variance, respectively, but both were not significant together. The effects of test scores and standard deviations on the reliability estimates have also been reported in other reliability generalizability studies (Blázquez-Rincón et al., 2022; Liang et al., 2021; López-Pina et al., 2015). Consistent with classical test theory, which posits that greater variation in observation scores enhances reliability, our findings align with previous studies showing that the variability in test scores influences reliability estimates (Vacha-Haase, 1998).

The present study identified reliability-induction issues associated with both the BFAS and BSMAS. This phenomenon can be attributed, in part, to a misunderstanding regarding the nature of reliability—specifically, whether it pertains to the measurement instrument itself or the outcomes derived from the testing process. Our findings underscore a prevalent misconception that reliability is an intrinsic quality of the testing tool, rather than a characteristic that is contingent upon specific testing conditions and sample populations. It is important to note that the reliability of psychological assessments is not an inherent property of the instrument; rather, it is a feature of the results obtained from the test. Consequently, administering the same assessment to different sample groups will inevitably yield varying reliability estimates due to factors such as differences in research samples, testing environments, cultural contexts, and linguistic backgrounds. Therefore, it is imperative for researchers to consistently report the reliability of a testing instrument as it pertains to their specific study context.

This study had certain limitations. The main limitations were as follows: Firstly, the research was restricted to English and Chinese publications, which could potentially limit the broader applicability of the findings. Secondly, there was a notable underrepresentation of studies from South America and Africa, and most studies lacked racial demographic data, which might restrict the thoroughness of the analysis. Thirdly, since race was not reported in the majority of studies, it was not included as a variable in this analysis. Fourthly, the exploratory model indicated that variations in test scores were the primary source of error. Nevertheless, a significant portion of the variations remained unexplained. Future research should consider including studies from a wider range of languages and regions. It should also explore the influence of racial and cultural factors, as well as investigate other possible factors that could moderate reliability.

The widespread use of social media has led to a prevalent issue of addiction, making the assessment of social media addiction a hot topic in the field of cyberpsychology. Effectively evaluating social media addiction is crucial for guiding adolescents to use social media responsibly and for intervening in cases of addiction. This paper employs the generalizability theory to conduct reliability analyses on the widely used BFAS scale and its variants, explores the sources of reliability variation, and further clarifies and confirms that the scale demonstrates high reliability. It is therefore suitable for a broad application in assessing social media addiction and can be used in clinical settings to identify participants who meet the criteria for addiction.

## 5 Conclusion

In summary, this pioneering study offers the first generalized assessment of the internal consistency reliability of the BFAS and BSMAS. Our results demonstrate that both tools have average reliability estimates exceeding 0.8, confirming their stability and dependability as evaluation instruments for social media addiction. This robust reliability underscores their suitability for use in a wide range of research and clinical settings. However, the study’s limitations, such as language and regional constraints, should be considered. Future research should aim to validate these findings in diverse languages and contexts, and examine additional factors that may impact the reliability of these tools.

## Data Availability

The original contributions presented in the study are included in the article/supplementary material, further inquiries can be directed to the corresponding author.
